# Structural Basis for Recognition of Cellular and Viral Ligands by NK Cell Receptors

**DOI:** 10.3389/fimmu.2014.00123

**Published:** 2014-03-26

**Authors:** Yili Li, Roy A. Mariuzza

**Affiliations:** ^1^W. M. Keck Laboratory for Structural Biology, Institute for Bioscience and Biotechnology Research, University of Maryland, Rockville, MD, USA; ^2^Department of Cell Biology and Molecular Genetics, University of Maryland, College Park, MD, USA

**Keywords:** NK receptor, MHC, virus, KIR, Ly49, NKG2, structure

## Abstract

Natural killer (NK) cells are key components of innate immune responses to tumors and viral infections. NK cell function is regulated by NK cell receptors that recognize both cellular and viral ligands, including major histocompatibility complex (MHC), MHC-like, and non-MHC molecules. These receptors include Ly49s, killer immunoglobulin-like receptors, leukocyte immunoglobulin-like receptors, and NKG2A/CD94, which bind MHC class I (MHC-I) molecules, and NKG2D, which binds MHC-I paralogs such as the stress-induced proteins MICA and ULBP. In addition, certain viruses have evolved MHC-like immunoevasins, such as UL18 and m157 from cytomegalovirus, that act as decoy ligands for NK receptors. A growing number of NK receptor–ligand interaction pairs involving non-MHC molecules have also been identified, including NKp30–B7-H6, killer cell lectin-like receptor G1–cadherin, and NKp80–AICL. Here, we describe crystal structures determined to date of NK cell receptors bound to MHC, MHC-related, and non-MHC ligands. Collectively, these structures reveal the diverse solutions that NK receptors have developed to recognize these molecules, thereby enabling the regulation of NK cytolytic activity by both host and viral ligands.

## Introduction

Natural killer (NK) cells are essential components of the innate immune response against viral infections and tumors (1–5). They not only eliminate virally infected or malignantly transformed cells by means of their cytolytic capabilities, but also produce cytokines and chemokines such as interferon-γ that modulate immune responses and help maintain tissue homeostasis. To perform these diverse functions, NK cells express a multitude of activating and inhibitory receptors that act in concert to regulate their activities (6, 7). NK receptors belong to two distinct structural families: the immunoglobulin (Ig) superfamily and the C-type lectin superfamily. In humans, NK receptors of the Ig superfamily are encoded in the leukocyte receptor complex (LRC) on chromosome 19 (7 in mouse) (8) and the NK gene complex (NKC) on chromosome 12 (6 in mouse) (9). Both superfamilies include inhibitory and activating receptors. In addition, NK receptors have been shown to recognize both cellular and viral ligands, including major histocompatibility complex (MHC), MHC-like, and non-MHC molecules.

The cytolytic activity of NK cells is regulated by positive signaling activating receptors (resulting in target cell lysis) and negative signaling inhibitory receptors (preventing lysis). It is the dynamic interplay between these signals that ultimately determines the outcome of NK cell–target cell encounters (4, 6, 7). The dominant signal received by an NK cell is inhibitory, provided by the interaction of its receptors with normal levels of MHC class I (MHC-I) molecules. If MHC-I expression is reduced by infectious or tumorigenic processes, this inhibitory signal is attenuated and the NK cell undergoes activation. As a consequence, cells with reduced MHC-I expression become subject to lysis by NK cells (1–5). The process by which NK receptors direct the cytolytic activity of NK cells against virally infected or tumor cells that have lost MHC-I expression is known as “missing-self” recognition.

Several receptor families on primate and rodent NK cells are responsible for monitoring MHC-I expression on surrounding cells (2–5, 10–13). These include the killer immunoglobulin-like receptors (KIRs) in humans, members of the Ly49 family (Ly49s) in rodents, NKG2/CD94 receptors, and leukocyte immunoglobulin-like receptors (LILRs). Although most Ly49s and KIRs inhibit NK function on binding to MHC-I ligands, some are activating (6, 7). Furthermore, the activating NK receptor NKG2D binds paralogs of MHC-I molecules, including MICA and RAE-I that are selectively upregulated in stressed tissues (14). The interaction of activating Ly49s with MHC-like proteins encoded by mouse cytomegalovirus (MCMV) has demonstrated a direct role for Ly49 receptors in anti-viral immunity (15–17).

Besides receptors specific for MHC-I or MHC-related ligands, a number of other receptors that recognize non-MHC proteins are involved in regulating NK cell cytotoxic activity (2, 7). These include CD16 (18), CD69 (19), NKR-P1 (20, 21), NTB-A (22, 23), 2B4 (22–24), DNAM-1 (25), NKp30 (26), NKp44 (27), NKp46 (28), NKp65 (21, 29), and NKp80 (21, 30, 31), which contribute to NK cell activation, and the inhibitory receptors, killer cell lectin-like receptor G1 (KLRG1) (32, 33) and LAIR-1 (34). The biological ligands for most (but not all) of these receptors have now been identified, including IgG Fc for CD16, Clr for NKR-P1, CD48 for 2B4, CD155 for DNAM-1, B7-H6 for NKp30, keratinocyte-associated C-type lectin (KACL) for NKp65, AICL for NKp80, E-cadherin for KLRG1, and collagen for LAIR-1 (2, 7).

Considerable progress has been made over the past few years in determining crystal structures of representative NK receptors, both in isolation and bound to MHC, MHC-related, or non-MHC ligands. These include both Ig-like (e.g., KIRs, LILRs, NKp30) and C-type lectin-like (e.g., Ly49s, NKG2D, NKG2/CD94, NKp65) receptors. These structures have revealed the multiplicity of solutions that NK receptors have evolved to recognize MHC, MHC-like, and non-MHC molecules, which collectively mediate crucial interactions for regulating the cytolytic activity of NK cells by host and viral ligands.

## MHC-I Recognition by KIR Receptors

The highly polymorphic KIR receptor family encodes the main MHC-monitoring molecules on primate NK cells and includes both inhibitory and activating members. KIRs are transmembrane glycoproteins containing two (D1 and D2) or three (D0, D1, and D2) extracellular C2-type Ig-like domains (10, 12). KIRs with two Ig-like domains are designated KIR2D; KIRs with three Ig-like domains are designated KIR3D. Whereas KIR2D receptors bind HLA-C alleles, KIR3D receptors bind HLA-A and HLA-B alleles. Crystallographic studies of KIR2D molecules, both in free form (35–39) and bound to HLA-C ligands (40, 41), have provided a framework for understanding the specificity of KIR2D receptors for HLA-C at the atomic level. In addition, the structure of KIR3DL1 in complex with HLA-B*5701 has revealed the basis for HLA recognition by three-domain KIRs (42).

**Figure 1:**
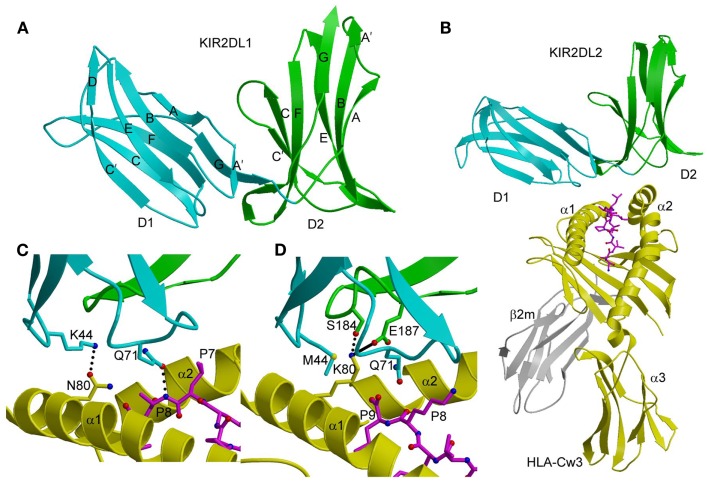
**Three-dimensional structures of KIR2DL and KIR2DL–HLA-C complexes**. **(A)** Ribbon diagram of KIR2DL1 (PDB accession code 1NKR). The D1 domain is cyan; D2 is green. The secondary structural elements are labeled. **(B)** Ribbons diagram of KIR2DL2 bound to HLA-Cw3 (1EFX). The α1, α2, and α3 domains of the HLA-Cw3 heavy chain are yellow; β_2_m is gray; the peptide is magenta. **(C)** Basis for allelic specificity and peptide selectivity of KIR2D receptors. The dotted lines represent hydrogen bonds formed by Asn80 of HLA-Cw3 with Lys44 of KIR2DL2, and by Gln71 of HLA-Cw3 with P8 of the peptide. **(D)** Interactions of Lys80 of HLA-Cw4 (yellow) with specificity-determining residues of KIR2DL1 (D1 domain in cyan, D2 domain in green) in the KIR2DL1–HLA-Cw4 complex (1IM9). The solid line represents a salt bridge.

The two N-terminal domains (D1 and D2) of KIR2D receptors are linked by a short hinge segment of three to five amino acids (Figure 1A). These Ig-like domains are each formed by two anti-parallel β-sheets, such that a β-sheet of four (in D1) or three (in D2) anti-parallel strands (ABED and ABE, respectively) juxtaposes a β-sheet of four anti-parallel strands (CC′ FG). The relative disposition of D1 and D2 is similar to that found in hematopoietic receptors (43, 44), with the angle between D1 and D2 ranging from 60° to 80° in different KIRD2 receptors.

In both the KIR2DL2–HLA-Cw3 (40) and KIR2DL1–HLA-Cw4 (41) complexes, the KIRs engage HLA-C through the α1 and α2 helices of the α1/α2 platform domain and the C-terminal portion of the MHC-bound peptide, with the D1–D2 axis orthogonal to the axis of the peptide (Figure 1B). This docking mode roughly resembles the way T-cell receptors (TCRs) bind MHC, but is completely distinct from the docking mode of LILR and Ly49 NK receptors (see below). Each KIR2D binds HLA-C using six loops from D1 and D2, which contact the α1 and α2 helices of the MHC-I molecule, respectively.

The structures of the KIR2DL2–HLA-Cw3 and KIR2DL1–HLA-Cw4 complexes explain the allelic specificity of KIR2DLs (40, 41). Of 12 HLA-Cw3 residues in contact with KIR2DL2, 11 are invariant in HLA-Cw4 and in all other HLA-C alleles. The only exception is Asn80, which defines the allelic specificity of KIR2DLs. Similarly, on the receptor side of the interface, 14 of 16 KIR2DL2 residues that contact HLA-Cw3 are conserved in KIR2DL1. The two exceptions are at positions 44 and 70. In the KIR2DL2–HLA-Cw3 structure, KIR2DL2 Lys44 makes a hydrogen bond with HLA-Cw3 Asn80; this hydrogen bond cannot be formed with KIR2DL1 Met44 (Figure 1C). In the KIR2DL1–HLA-Cw4 structure, the side chain of HLA-Cw4 Lys80 is situated in a negatively charge pocket of KIR2DL1 that includes Met44, which contacts HLA-Cw4 Lys80 (Figure 1D). Replacement of Met44 by lysine, as in KIR2DL2, would cause charge repulsion with HLA-Cw4 Lys80, resulting in loss of binding.

KIR2DS4 is an activating receptor that specifically recognizes HLA-A*11, as well as HLA-C allotypes bearing the C1 and C2 epitopes (39). A comparison of the unbound KIR2DS4 structure with the KIR2DL2–HLA-Cw3 and KIR2DL1–HLA-Cw4 complexes revealed two features that likely explain the binding specificity of KIR2DS4. First, a backbone displacement of one of the predicted HLA-contacting loops (L2) of KIR2DS4, relative to its position in KIR2DL2, may disrupt the interaction between Lys44 of KIR2DS4 and Asn80 of the HLA-C C1 epitope, resulting in weaker binding of KIR2DS4 to C1^+^ allotypes than KIR2DL2 (39). Conversely, this displacement could increase avidity for C2^+^ allotypes by accommodating Lys80 of the C2 epitope. Second, the Pro71–Val72 motif of KIR2DS4 that confers reactivity with HLA-A*11 is part of a loop (L3), which in KIR2DL2 contacts HLA-C using the Gln71–Asp72 motif (40). Replacement of Gln71–Asp72 by Pro71–Val72 in KIR2DS4, which is the result of gene conversion with KIR3DL2, reduces avidity for C1^+^ allotypes but increases avidity for HLA-A*11 and C2^+^ allotypes (39).

The binding of KIR2D receptors to HLA-C molecules displays preferences for certain peptides (45, 46); however, whether peptide selectivity has a role in NK receptor function is not clear. Intriguingly, KIR-associated HIV-1 sequence polymorphisms in chronically infected individuals have been found to increase the binding of inhibitory KIRs to CD4^+^ T cells infected with HIV-1, and to decrease the anti-viral activity of KIR-positive NK cells (47). Consistent with the observation that the KIR binding site is centered near C-terminal residues P7 and P8 of the MHC-bound peptide (40, 41), the KIR–HLA interaction is most sensitive to substitutions at these two peptide positions. By contrast, TCRs, which exhibit much greater peptide specificity than KIRs, typically focus on the central P5 position of the peptide (48). Since the peptide positions recognized by KIRs are not usually directly involved in TCR binding, MHC molecules may be able to evolve their polymorphic regions to present diverse microbial (i.e., foreign) peptides for T-cell-mediated immunity, while at the same time maintaining non-variant regions to bind self-peptides for KIR recognition and NK-cell-mediated immune defense.

**Figure 2:**
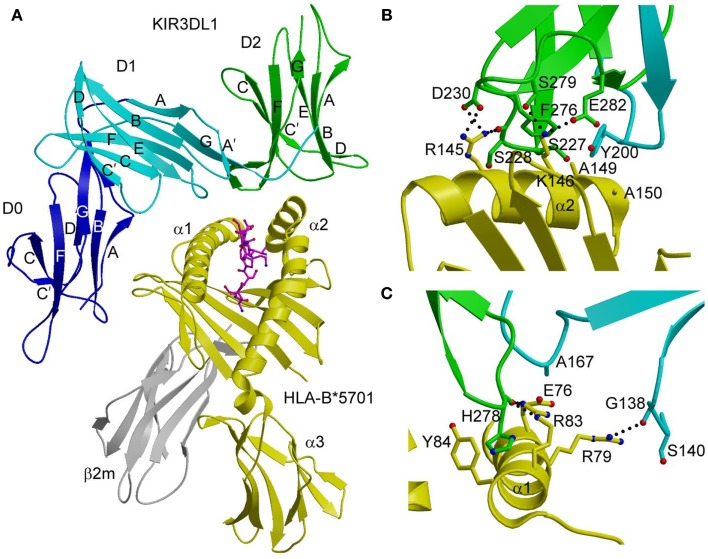
Structure of the KIR3DL1–HLA-B*5701 complex. **(A)** Ribbon diagram of KIR3DL1 bound to HLA-B*5701 (3VH8). The orientation of the MHC-I ligand is similar to that of HLA-Cw3 in the KIR2DL2–HLA-Cw3 complex (Figure 1B). The HLA-B*5701 heavy chain is yellow; β_2_m is gray; the peptide is magenta. The KIR3DL1 D0 domain is dark blue; D1 is cyan; D2 is green. The secondary structural elements of KIR3DL1 are labeled. **(B)** Contacts between KIR3DL1 and the HLA-B*5701 α2 helix. The D2 domain mainly interacts with HLA-B*5701 residues 142–151, which display limited polymorphism among HLA-B alleles. At the center of the D2–HLA-B*5701 interface, KIR3DL1 residues Tyr200 and Phe276 form an aromatic cluster that converges on the α2 helix. **(C)** Contacts between KIR3DL1 and the HLA-B*5701 α1 helix. KIR3DL1 recognizes HLA allotypes that contain the Bw4 epitope-defining residues 77–83 on the α1 helix, which likely accounts for the allelic specificity of KIR3DLs.

In the KIR3DL1–HLA-B*5701 complex (42), KIR3DL1 engages HLA-B*5701 with its D1 and D2 domains situated over the C-terminal half of the peptide-binding groove in an overall orientation highly similar to that of the KIR2D–HLA-C complexes (Figure 2A). KIR3DL1 adopts an elongated conformation, which allows D0 to extend down toward β_2_-microglobulin (β_2_m) and engage a region of the MHC-I molecule that is nearly invariant across HLA-A and HLA-B allotypes. The D1 domain contacts the α1 helix and the self-peptide, while the D2 domain contacts the α2 helix. The D2 domain mainly interacts with HLA-B*5701 residues 142–151, which display restricted polymorphism across HLA-B alleles. At the center of the D2–HLA-B*5701 interface, KIR3DL1 residues Tyr200 and Phe276 form an aromatic cluster that converges on the α2 helix (Figure 2B). Alanine substitution of these residues abrogated binding to HLA-B*5701, demonstrating the importance of this central core to HLA recognition.

KIR3DL1 recognizes HLA allotypes that contain the Bw4 epitope, which is defined by residues 77–83 of the α1 helix. In the structure (42), KIR3DL1 contacts residues 79, 80, and 83 within the Bw4 epitope through its D1 domain (Figure 2C), which likely accounts for the allelic specificity of KIR3DLs. The D1 domain also makes limited contacts with the self-peptide at position P8, analogous to the interaction of KIR2D receptors with peptides bound to HLA-C (see above).

Unexpectedly, the extensive polymorphisms found within individual KIR3D families are located predominantly at positions not implicated in HLA binding. This implies that most KIR3D polymorphisms, a number of which are subject to positive selection (49), are unlikely to impact affinity directly, but could potentially affect HLA binding indirectly by altering the clustering or expression levels of KIR3D receptors on the NK cell surface. In this way, evolutionary pressures may drive the diversification of KIR3D sequences at sites remote from the HLA-binding site.

## MHC-I Recognition by LILRs

The human LILR family of immunoreceptors (also referred to as Ig-like transcripts, or ILTs) is broadly expressed on NK cells, T cells, monocytes, B cells, and dendritic cells (50). The mouse orthologs of LILRs are known as paired immunoglobulin receptors (PIRs). Like KIRs, LILR receptors contain either two or four tandem extracellular Ig-like domains. LILRA1, LILRA2, LILRA3, LILRB1, and LILRB2 bind classical MHC-I proteins (HLA-A, -B, and -C), whereas LILRA4, LILRA5, LILRA6, LILRB3, LILRB4, and LILRB4 do not appear to recognize MHC-I. The inhibitory LILRB1 and LILRB2 receptors bind multiple MHC-I molecules, both classical and non-classical (HLA-E, -F, and -G), with comparable kinetics and affinities (51, 52). By contrast, individual KIR receptors display allelic specificity, as discussed above. In addition to their role as MHC-I sensors, LILRs may be involved in immune responses to viral infections, as suggested by the finding that LIRLB1 is a receptor for UL18 (53). This immunoevasin is an MHC-I homolog encoded by human cytomegalovirus (HCMV). The crystal structure of LILRB1 (domains D1 and D2 only) has been solved in free form (54) and bound to HLA-A2 (55) and UL18 (56). Structures have also been reported for LILRB2 (D1 and D2) in unbound form (57) and in complex with HLA-G (58).

**Figure 3:**
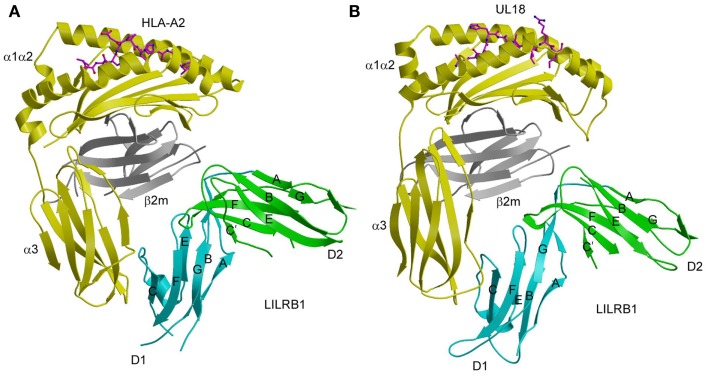
**Interaction of LILRB1 with MHC-I and a viral MHC-I mimic**. **(A)** Structure of LILRB1 bound to HLA-A2 (1P7Q). The α1, α2, and α3 domains of the HLA-A2 heavy chain are yellow; β_2_m is gray; the peptide is magenta. The D1 and D2 domains of LILRB1 are colored in cyan and green, respectively. The secondary structural elements of LILRB1 are labeled. **(B)** Structure of LILRB1 bound to the HCMV MHC-I mimic UL18 (3D2U).

Similar to KIR2D (Figure 1A), the two tandem Ig-like domains of both LILRB1 and LILRB2 form a bent structure characterized by an acute interdomain angle (Figure 3A). Each domain comprises two anti-parallel β-sheets arranged in a topology like that of KIRs. In the LILRB1–HLA-A2 complex (55), LILRB1 D1D2 binds the side of HLA-A2, forming two contact surfaces that include residues from β_2_m, which is invariant, and the HLA-A2 α3 domain, which is relatively non-polymorphic. The D1–D2 interdomain hinge region contacts β_2_m, while the tip of LILRB1 D1 contacts the HLA-A2 α3 domain, (Figure 3A). Similar to LILRB1, LILRB2 recognizes β_2_m and the HLA-G α3 domain using the interdomain hinge and D1, respectively (58). The docking mode utilized by LILRB1 and LILRB2, which differs completely from that of KIRs (Figure 1B), is consistent with MHC-I recognition in a peptide-independent manner. The focus by LILRs on conserved elements of MHC-I molecules, both classical and non-classical, accounts for the broad specificity of these NK receptors for numerous HLA alleles.

Whereas LILRB1 undergoes an interdomain angle change of ~15° after binding MHC-I, LILRB2 maintains the same interdomain angle (55, 58). Overall, however, LILRB2 exhibits greater conformational changes than LILRB1 upon complex formation. In particular, free LILRB2 contains only one 3_10_ helix (residues 52–55) involving binding site residues, whereas bound LILRB2 contains two such helices in the interface with HLA-G (residues 46–50 and 53–57). By contrast, LILRB1 contains two 3_10_ helices in both free and bound states. Although affinity measurements indicate that the membrane-distal D1 and D2 domains are mainly responsible for HLA binding (54, 55), a role for the membrane-proximal D3 and D4 domains cannot be formally excluded in the absence of a structure of a four-domain LILR in complex with ligand. A complete D1–D4 LILR structure is also required to understand the apparent ability of LILR/PIR receptors to bind not only MHC-I molecules on opposing cells (*trans* interaction), but also ones on the same cell (*cis* interaction) (59, 60), as discussed below.

## LILR Recognition of UL18, a Viral MHC-I Mimic

Among the microorganisms that have achieved great success in inventing strategies for immune evasion are the cytomegaloviruses, whose genomes encode proteins that interfere with both NK cell and T-cell recognition, as well as antigen processing and presentation (61–63). These include proteins that are known, or predicted to be, structural homologs of host MHC-I molecules. HCMV encodes an MHC-I homolog, UL18, that binds the inhibitory receptor LILRB1 (64). This interaction is believed to allow HCMV-infected cells to avoid NK-cell-mediated lysis (65). UL18 is a heavily glycosylated transmembrane protein that associates with β_2_m, and with endogenous peptides derived from host cytoplasmic proteins that resemble those bound to HLA alleles (66). Remarkably, UL18 binds LILRB1 >1000-fold more tightly than MHC-I proteins, enabling this decoy ligand to compete effectively with MHC-I for binding to LILRB1 (67).

Despite sharing only ~25% sequence with its MHC-I counterparts, the structure of UL18 bound to LILRB1 shows striking similarity to the LILRB1–HLA-A2 and LILRB2–HLA-G complexes, with the tip LILRB1 D1 domain contacting the UL18 α3 domain and the D1–D2 interdomain hinge contacting β_2_m (Figure 3B) (56). Variable residues in the UL18 α1 domain, which were identified by sequence analysis of laboratory and clinical HCMV strains, do not contact LILRB1, although domains D3 and D4, which are not present in the structure, could potentially engage this region of UL18. Most contacts between LILRB1 and U18 involve the UL18-specific portion of the UL18/β_2_m heterodimer (i.e., the heavy chain), whereas the majority of LILRB1 interactions with HLA-A2 involve the invariant β_2_m light chain. Additional salt bridges and better surface complementarity in the LILRB1–UL18 interface compared with the LILRB1–HLA-A2 interface likely explain the >1000-fold higher affinity of UL18.

A major difference between UL18 and MHC-I molecules is the exceptionally high carbohydrate content of UL18, which is attributable to its 13 potential N-glycosylation sites, compared to only one *N*-glycan attached to human MHC-I molecules. In fully glycosylated UL18 (the protein used for crystallization was minimally glycosylated), most of the surface of UL18 was predicted to be covered by carbohydrate, with the notable exceptions of the binding site for LILRB1 and the docking interface with β_2_m (56). This suggests that UL18 evolved a glycan shield to prevent neutralization by antibodies, while preserving the binding site for LILRs. Such a strategy for reducing immunogenicity is analogous to that employed by other viruses with heavily glycosylated envelope proteins, notably HIV and influenza (68).

## Natural Cytotoxicity Receptors

Natural cytotoxicity receptors (NCRs) were discovered in a search for receptors that activated NK cells independently of MHC (69). To date, the NCR family includes NKp30 (NCR3, CD337), NKp44 (NCR2, CD336), and NKp46 (NCR1, CD335). In humans, NKp44 and NKp30 are encoded in the class III region of the MHC locus, while NKp46 is encoded in the LRC (69). Mice only possess a functional gene for NKp46. These very potent activating receptors comprise one (NKp30 and NKp44) or two (NKp46) Ig-like extracellular domains (69, 70). NCRs contain charged residues in their transmembrane regions for association with immunoreceptor tyrosine-based activation motif (ITAM)-bearing signaling polypeptides: ζ–γ for NKp30 and NKp46; and DAP12 for NKp44 (71). In humans, NCRs play a major role in NK-cell-mediated lysis of diverse tumor cells, including carcinomas, neuroblastomas, and leukemias (69, 70). In addition, NCRs have been implicated in protective responses against various viruses, including influenza (72), hepatitis C (73), West Nile (74), and Ebola (75).

Despite intensive efforts over many years, ligands for the NRC family have proven very elusive and, in some cases, controversial. NKp44 and NKp46 bind influenza and other viral hemagglutinins (HAs) mainly through recognition by the HA of terminal sialic acid moieties (the cellular receptor for HAs) on N-linked glycans of these NCRs (72, 76, 77). Although this mechanism would allow NKp44 and NKp46 to bind a wide variety of viruses, due to the ability of HAs to bind sialic acid-containing glycoproteins in general, this is probably not the full story, since recognition would not depend on the NCR ectodomain itself, but only on the fact that NKp44 and NKp46 are glycoproteins with terminal sialic acids (13). Binding of NKp46 to heparan sulfate proteoglycans has also been described (78), but the biological relevance of this interaction is unclear. Recently, a novel isoform of the mixed-lineage leukemia-5 protein (MLL5) was identified as a cellular ligand for NKp44 (79). This MLL5 isoform was not expressed on cells from healthy individuals, but was detected on a large panel of tumor and transformed cell lines. Moreover, MLL5 expression on target cells triggered NKp44-mediated NK cell cytotoxicity.

NKp30 binds the nuclear factor BAT3 (80) and the tumor cell surface protein B7-H6 (81). BAT3 (also known as BAG-6) has been implicated in the induction of apoptosis after endoplasmic reticulum stress or DNA damage (82). B7-H6 is a member of the B7-family (81), which includes ligands (B7-1 and B7-2) for the T-cell co-inhibitory receptor CTLA-4 and the co-stimulatory receptor CD28 (83). The B7-family also encodes PD-L1 and PD-L2, which are ligands for the T-cell co-inhibitory receptor PD-1. B7-H6 is not expressed in normal human tissues, but can be detected on a variety of human tumor cell lines that includes T and B lymphomas, melanomas, and carcinomas (81). Importantly, B7-H6 expression on tumor cells triggered NK cell cytotoxicity that was mediated specifically by NKp30. These results implicate B7-H6 as tumor-induced self-protein, analogous to MICA (2), which alerts NK cells to cellular transformation (81). NKp30 also recognizes the tegument pp65 protein of HCMV, indicating a role for this NCR in anti-viral immunity (84). Recently, NKp30 was shown to be responsible for the recognition and killing of the opportunistic fungi *Cryptococcus* and *Candida* (85). Although the fungal ligand recognized by NKp30 remains to be identified, possible candidates include β-1,3 glucans, which are major components of fungal cell wells and are highly conserved across fungal species. Thus, NKp30 interacts with multiple ligands, as do the activating NK receptors NKG2D and DNAM-1 (86, 87).

**Figure 4:**
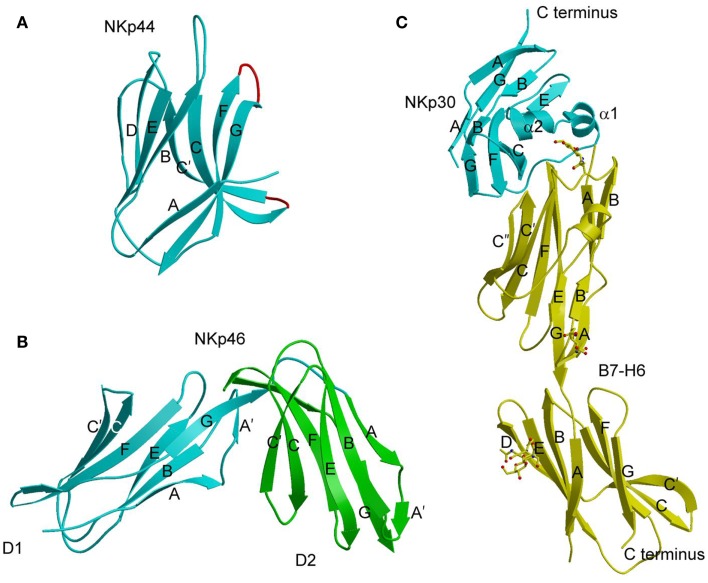
**Natural cytotoxicity receptors**. **(A)** Structure of NKp44 (1HKF). The β-strands are labeled. The CC′ and FG loops, drawn in red, define a positively charged surface groove that may serve as a binding site for anionic ligands. **(B)** Structure of NKp46 (1P6F). D1 is cyan; D2 is green. **(C)** Structure of NKp30 bound to its tumor cell ligand B7-H6 (3PV6). N-linked glycans at B7-H6 residues Asn43 and Asn57 in the V-like domain and Asn208 in the C-like domain are shown in ball-and-stick representation.

At present, crystal structures have been determined for NKp30, NKp44, and NKp46 in unbound form (88–90), and for NKp30 bound to B7-H6 (91). NKp44 comprises a single V-type Ig-like domain that features a prominent groove formed by two facing β-hairpin loops (CC′ and FG) projecting from the Ig fold core (Figure 4A) (88). The solvent accessibility of the groove, and its electropositive nature, suggest a possible binding site for anionic ligands, such as sialic acid, although no structure of a complex has been reported. NKp46 consists of two C2-set Ig-like domains whose overall fold and disposition are similar to those of the D1D2 domains of KIRs and LILRs (Figure 4B) (89). This structural resemblance suggests that similar receptor surfaces may be involved in ligand binding. The region of NKp46 analogous to the KIR or LILR ligand-recognition site is located at the interdomain hinge and comprises residues from both Ig-like domains. However, confirmation of this hypothesis awaits structural studies of NKp46–ligand complexes.

The Ig-like domain of NKp30 exhibits the chain topology found in C1-set domains (Figure 4C) (90, 91). The closest structural homolog of NKp30 is PD-L1, a ligand for PD-1. Like PD-1, NKp30 is a member of the CD28 family, which also includes CTLA-4, ICOS, and B and T lymphocyte attenuator (BTLA) (81). Similar to other B7-family members, the extracellular portion of B7-H6 consists of a V-like and a C-like domain, with the V-like domain distal from the membrane (91).

**Figure 5:**
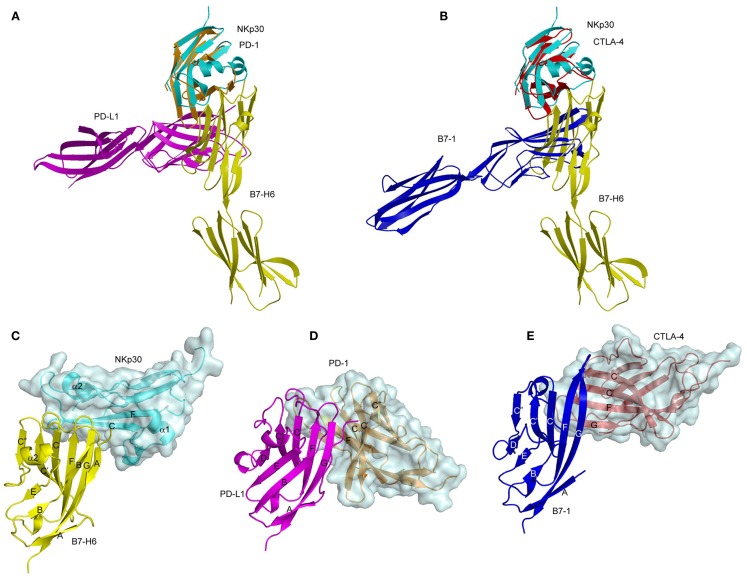
**Comparison of the NKp30–B7-H6, PD-1–PD-L1, and CTLA-4–B7-1 complexes**. **(A)** Overlay of the NKp30–B7-H6 (3PV6) and PD-1–PD-L1 (3BIK) complexes by superposing NKp30 (cyan) onto PD-1 (brown). B7-H6 is yellow; PD-L1 is violet. **(B)** Overlay of the NKp30–B7-H6 and CTLA-4–B7-1 (1I8L) complexes by superposing NKp30 (cyan) onto CTLA-4 (red). B7-H6 is yellow; B7-1 is dark blue. **(C–E)** Docking modes in the NKp30–B7-H6, PD-1–PD-L1, and CTLA-4–B7-1 complexes. The three complexes were overlaid by superposing the IgV domains of B7-H6 (yellow), PD-1-L1 (violet), and B7-1 (dark blue), then translated horizontally for viewing.

The structure of the NKp30–B7-H6 complex revealed a binding interface formed by the front β-sheet of the B7-H6 V-like domain and the front and back β-sheets of the NKp30 C-like domain (Figure 4C) (91). The overall architecture of the NKp30–B7-H6 complex differs considerably from those of the PD-1–PD-L1 (or PD-1–PD-L2) (92, 93) and CTLA-4–B7-1 (or CTLA-4–B7-2) complexes (94, 95), as is evident from superposing these complexes (Figures 5A,B). Relative to NKp30, PD-1 and CTLA-4 bind their ligands at angles of ~90° and ~60°, respectively. Whereas the PD-1–PD-L2 and CTLA-4–B7-1 interfaces are dominated by strand-to-strand interactions (Figures 5D,E), B7-H6 engages NKp30 in an antibody-like manner, with greater involvement by the loops of the B7-H6 V-like domain (Figure 5C). Thus, the protruding FG loop of B7-H6, which corresponds to complementarity-determining region (CDR) 3 of antibodies, fits into a deep groove on NKp30, with additional contacts provided by the BC (CDR1-like) and C′C′′(CDR2-like) loops of B7-H6.

Besides B7-H6, NKp30 recognizes the tegument pp65 protein of HCMV (84) and the nuclear factor BAT3 (80). Remarkably, B7-H6, pp65, and BAT3 appear completely unrelated, both in terms of three-dimensional structure and origin (B7-H6 is a host surface protein; pp65 is a viral structural protein; BAT3 is a host nuclear protein). How NKp30, a relatively small receptor comprising a single Ig-like ectodomain, can bind such disparate ligands is at present a mystery.

## MHC-I Recognition by Ly49 Receptors

The highly polymorphic Ly49 receptors are the principle MHC-monitoring molecules on rodent NK cells. In mice, the Ly49 family includes at least 23 members (Ly49A–W), along with multiple allelic variants (96, 97). Most Ly49s inhibit NK-cell-mediated cytolysis upon recognizing one or more H-2D or H-2K alleles (96, 98, 99). However, some Ly49s are activating. In general, Ly49s recognize MHC-I independently of the bound peptide, although Ly49C and Ly49I display considerable peptide specificity (100, 101).

Like NKG2D and NKG2/CD94, Ly49 receptors are members of the C-type lectin-like family of proteins (10, 11). However, none of these NK receptors have a functional calcium-binding site. Ly49s are homodimeric type II transmembrane proteins (N-terminus inside the NK cell), with each chain containing a C-type lectin-like domain (CTLD), known as the natural killer receptor domain (NKD). Each NKD of the Ly49 homodimer is linked by a stalk of ~70 residues to the transmembrane and cytoplasmic domains. Activating and inhibitory receptors differ with regards to their cytoplasmic domains: whereas inhibitory Ly49s transduce signals via immunoreceptor tyrosine-based inhibitory motifs (ITIM), activating Ly49s instead use the associated signaling homodimer DAP12, which possesses an ITAM (96, 98).

Extensive structural information is available for Ly49 receptors in MHC-bound and unbound forms. Crystal structures have been reported for Ly49A NKD in complex with H-2D^d^ (102), Ly49C NKD bound to H-2K^b^ (103, 104), Ly49C NKD (104), Ly49I NKD (105), Ly49G2 NKD (104), Ly49L NKD (106), Ly49L NKD with the stalk region (106), and Ly49H bound to the MCMV immunoevasin m157 (107). Together, these structures have revealed the molecular architecture of the MHC-binding site of Ly49 receptors, the basis for MHC-I engagement in *trans* and *cis*, and the means by which viral immunoevasins target Ly49s.

**Figure 6:**
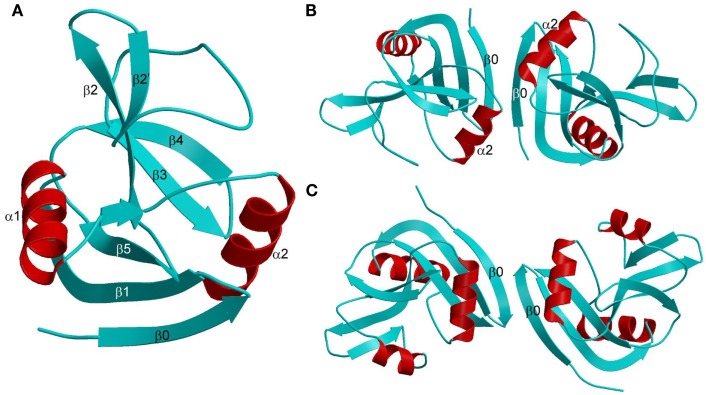
**Structures of Ly49 NK receptors**. **(A)** Ribbon drawing of the Ly49A C-type lectin-like domain (1QO3). Secondary structure elements are labeled. β-strands and loops are cyan; α-helices are red. **(B)** Structure of the “closed” Ly49A homodimer. Secondary structure elements that participate in formation of the dimer interface are labeled. The α2 helices are juxtaposed. **(C)** Structure of the “open” Ly49C homodimer (3C8J). The α2 helices do not make contact across the dimer interface.

The Ly49 NKD is composed of two α-helices, designated α1 and α2, and two anti-parallel β-sheets formed by seven β-strands (Figure 6A). Ly49 receptors exist exclusively as dimers on the NK cell surface. In the dimers, two NKDs associate through strand β0 to create an extended anti-parallel β-sheet. Ly49 dimers may adopt two distinct conformations: “closed” and “open,” as exemplified by Ly49A (Figure 6B) and Ly49C (Figure 6C), respectively. In the closed dimer, the C-terminal ends of the α2 helices are juxtaposed, whereas in the open dimer the α2 helices do not contact each other. As explained below, this variability in Ly49 dimerization geometry serves to modulate the way NK receptors bind MHC (102–104).

Although Ly49s and other C-type lectin-like NK receptors (e.g., NKG2D, NKG2/CD94, NKp65) retain a lectin-like fold, specific structural features required for lectin activity are absent, enabling these receptors to recognize proteins as opposed to carbohydrates. Most notably, C-type lectin-like NK receptors do not contain bound calcium ions due to missing calcium-coordinating residues.

**Figure 7:**
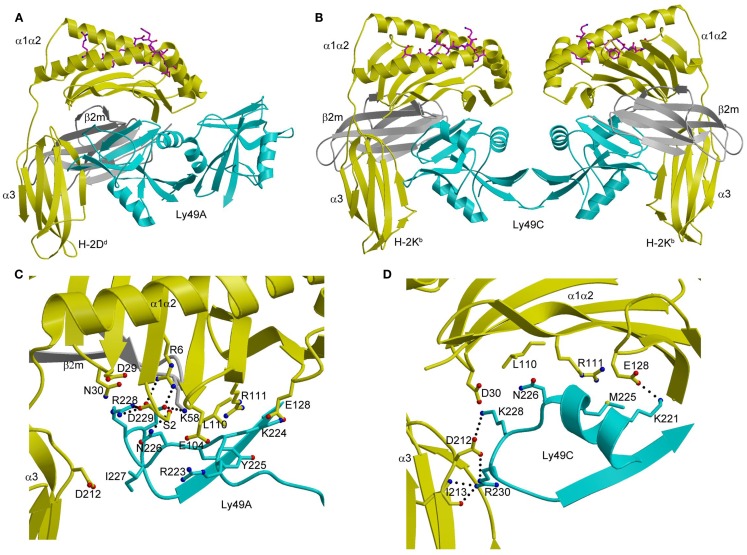
**Structures of Ly49–MHC-I complexes**. **(A)** Ribbon diagram of Ly49A bound to H-2D^d^ (1QO3). The α1, α2, and α3 domains of the MHC-I heavy chain are yellow; β_2_m is gray; the peptide is magenta; the Ly49A dimer is cyan. In this view, the complex is oriented with the H-2D^d^ molecule on the target cell at the bottom; the Ly49A homodimer reaches H-2D^d^ from an opposing NK cell at the top, to which it is connected via stalk regions projecting down to the N-termini of the subunits. **(B)** Structure of Ly49C in complex with H-2K^b^ (3C8K). **(C)** The Ly49A–H-2D^d^ interface, highlighting interactions made by residues 211–231 of Ly49A. **(D)** The Ly49C–H-2K^b^ interface, showing interactions made by the corresponding region of Ly49C. The side chains of interacting residues are drawn in ball-and-stick representation, with carbon atoms in cyan (Ly49A or Ly49C), yellow (H-2D^d^ or H-2K^b^), or gray (β_2_m), oxygen atoms in red, and nitrogen atoms in blue. Hydrogen bonds are represented by dotted lines.

In the Ly49A–H-2D^d^ complex (Figure 7A), the Ly49A homodimer engages a single H-2D^d^ molecule using only one of its subunits, at a site beneath the peptide-binding platform of the MHC-I ligand (102). This site partially overlaps the binding sites for CD8 and LILRB1 (Figure 3A). In the Ly49C–H-2K^b^ complex (103, 104), by contrast, the Ly49C dimer binds H-2K^b^ bivalently, with each subunit making identical contacts with MHC-I at a site equivalent to Ly49A binding site on H-2D^d^ (Figure 7B).

The different dimerization geometries of Ly49A and Ly49C account for the different modes of MHC engagement in the Ly49A–H-2D^d^ and Ly49C–H-2K^b^ complexes. The closed Ly49A dimer cannot simultaneously bind two MHC ligands, like the open Ly49C dimer, because this would result in major steric clashes between MHC molecules, at least in the crystal. However, an NMR study revealed that Ly49A exists predominantly as an open dimer in solution that can bind two MHC-I molecules (108). The most likely interpretation of the combined results from X-ray crystallography and NMR is that Ly49 receptors are present on the NK cell surface in dynamic equilibrium between an open form, which permits bivalent MHC binding, and a closed form, which only allows engagement of one MHC ligand.

As in the case of KIRs (see above), Ly49s display specificity for different MHC alleles. Thus, whereas the promiscuous Ly49C receptor recognizes H-2K^b^, H-2K^d^, H-2D^b^, H-2D^d^, and H-2D^k^, the more specific Ly49A and Ly49C receptors only bind H-2D^d^ and H-2D^k^ (97, 101). Ly49s have developed a two-tiered strategy for recognizing MHC, as deduced from the Ly49A–H-2D^d^ and Ly49C–H-2K^b^ structures (104). Primary recognition is carried out by a central region consisting of strand β3, loop L5, and strand β4 (residues 232–243). This central region has a conserved structure across Ly49s and contributes most to the binding energetics. Supplementing these primary interactions are secondary ones mediated by a region flanking the central region that comprises residues 218–231. This region, which exhibits high sequence variability across the Ly49 family, confers different MHC specificities. It adopts markedly different conformations in Ly49A (Figure 7C) and Ly49C (Figure 7D), and separates Ly49s into ones that recognize both H-2D and H-2K ligands (e.g., Ly49C) versus ones that only recognize H-2D (e.g., Ly49A) (104).

The finding that MHC recognition by Ly49A is independent of the sequence of the MHC-bound peptide in both cellular and binding assays is readily explained by the total absence of direct contacts between Ly49A and the peptide in the Ly49A–H-2D^d^ structure (Figure 7A) (102). By contrast, the remarkable peptide selectivity of Ly49C is difficult to understand in terms of the Ly49C–H-2K^b^ complex, in which there is also a complete lack of contacts between Ly49C and the peptide (Figure 7B) (103, 104). Although the potential biological role of the peptide selectivity of certain Ly49s (and KIRs) remains obscure, the description of a functional interaction between Ly49P and H-2D^k^ on MCMV-infected cells that confers resistance to the virus suggests an intriguing possibility (17). This interaction requires the participation of the MCMV gene product m04/gp34 (109). This virulence factor associates with MHC-I in the endoplasmic reticulum and travels to the cell surface (110). Although the molecular nature of the Ly49P–H-2D^k^ interaction on MCMV-infected cells remains to be defined, one possibility is that m04/gp34 provides a specific peptide recognized by Ly49P in an H-2D^k^-dependent manner, which would confer on NK cells a degree of specificity for viral pathogens reminiscent of that of cytotoxic T cells (11, 17, 109).

## *Trans* and *Cis* Interactions of Ly49 Receptors with MHC-I

Cell surface receptors mediate cell-to-cell communication by interacting with ligands expressed on other cells (*trans* interactions). In addition, some cell surface receptors have been shown to bind ligands expressed on the same cell via *cis* interactions (60, 111). These include the structurally unrelated NK receptors Ly49 and LILRs, which interact not only with MHC-I molecules on other cells in *trans*, but also with MHC-I molecules on the same cell in *cis* (59, 112, 113). Other examples of cell surface receptors that bind ligands in both *trans* and *cis* are: siglec-2 (CD22), a negative regulator of B cell receptor signaling that recognizes sialic acid modifications of glycoproteins (114–117); herpes virus entry mediator (HVEM), which is regulated by its ligand BTLA (118); plexin receptors that bind semaphorins (119); and notch receptors that bind Delta (120, 121). The emerging theme from these studies is that *cis* interactions regulate effector cell function by modulating (decreasing or increasing) the threshold at which cellular activation signals produce a biological response (60, 111).

*Cis* interactions between Ly49 receptors and MHC-I ligands facilitate NK cell activation (112). This effect is not the result of inhibitory signaling through constitutive ITIM phosphorylation of Ly49s. Rather, *cis* interactions with MHC-I sequester, or mask, Ly49s to render them physically unavailable for functional *trans* interactions (113). In this way, *cis* interactions lower the threshold at which NK cell activation exceeds inhibition, considerably increasing the sensitivity of NK cells to diseased cells (60). Remarkably, in addition to modulating inhibitory function, *cis* interactions of Ly49A are necessary for NK cell education (122). As in the case of Ly49, the interaction of LILRB/PIR-B receptors with MHC-I in *cis* is constitutive (59). However, unlike Ly49, the ITIMs of LILRB/PIR-B receptors are phosphorylated constitutively, such that *cis* binding generates tonic inhibitory signals that dampen NK cell activation.

**Figure 8:**
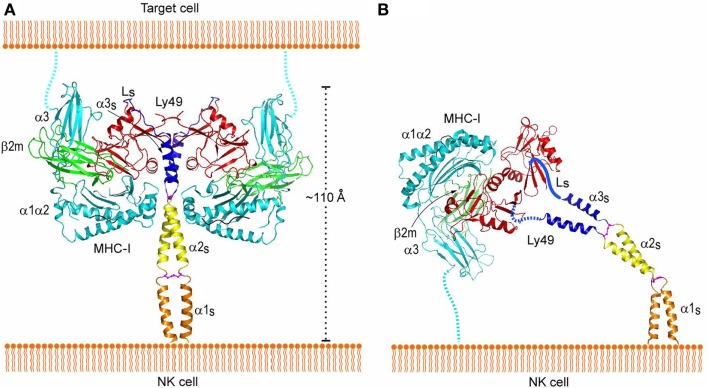
**Interaction of Ly49 receptors with MHC-I in *trans* and *cis***. **(A)** *Trans* interaction of an Ly49 receptor with two MHC-I molecules, based on the structures of Ly49L (3G8L) and the Ly49C–H-2K^b^ complex (3C8K). The α1, α2, and α3 domains of the MHC-I heavy chain are cyan; β_2_m is green; Ly49 NKD is red; helix α3_S_ of the Ly49 stalk and loop L_S_ connecting α3_S_ to the NKD are blue; the disulfide bond linking the α3_S_ helices is magenta. The predicted α1_S_ and α2_S_ helices of the stalk are orange and yellow, respectively, with the disulfide bond in magenta. The Ly49 homodimer on the NK cell binds two MHC-I molecules on the target cell. To bind in *trans*, the stalks must adopt a backfolded conformation, as the N-termini of the Ly49 monomers point away from the NK cell membrane (Ly49s are type II transmembrane proteins). **(B)** *Cis* interaction of Ly49 with MHC-I, based on the structure of Ly49L and the Ly49A–H-2D^d^ complex (1QO3). The L_S_ loops connecting the α3_S_ helices to the NKDs are drawn arbitrarily. The Ly49 homodimer binds one MHC-I molecule on the NK cell itself. In this case, the stalks must assume an extended conformation, as the N-termini of the Ly49 monomers point toward the NK cell. Reproduced with permission from *Immunity* (106).

*Trans* and *cis* interactions by Ly49 receptors utilize the same binding site beneath the peptide-binding platform of MHC-I (112). Therefore, Ly49s must drastically reorient their NKDs relative to the NK cell membrane in order to bind MHC-I in *trans* versus *cis*. Most likely, it is the exceptionally long stalk regions of Ly49s (~70 residues) that provide the requisite flexibility. In the crystal structure of Ly49L, which includes the C-terminal 40 residues of the stalk region (designated the α3s segment), the stalk is composed of an α-helix and a 12-residue loop linking the helix to the NKD (Figure 8A) (106). However, instead of projecting from the NKD, as is typical for a stalk region, the Ly49L stalk backfolds onto the NKD. In a modeled Ly49–MHC-I complex (Figure 8A), the N-termini of the stalk regions point in a completely opposite direction from the C-termini of the MHC-I molecules. Therefore, Ly49s likely adopt the backfolded conformation to bind MHC-I in *trans*. On the other hand, *cis* binding requires the stalks to assume an extended conformation that orients the N-termini of the NKDs toward the NK cell (Figure 8B). Unlike the *trans* interaction, where one Ly49 dimer engages two MHC-I ligands (Figure 3A), this model precludes *cis* engagement of both NKDs by MHC-I, because of the orientation that binding of one MHC-I ligand would impose on the Ly49 dimer (Figure 3B). In agreement with the model, biochemical analyses confirmed that *trans* binding of MHC-I by Ly49 dimers occurs in a bivalent fashion, whereas *cis* binding is monovalent (106). Moreover, Ly49 receptors appear able to switch between backfolded and extended conformations (108, 123).

*Cis*–*trans* binding may typically require major structural changes analogous to those of Ly49. However, the stalk regions of LILRB/PIR-B receptors are much shorter than those of Ly49. Accordingly, the ligand-binding D1 and D2 domains of LILRB would need to reverse direction relative to the surface of the NK cell in order to engage MHC-I in *cis*. To do so, LILRB would need to bend back on itself and assume a horseshoe-shaped arrangement of the four Ig domains (D1–D4), as observed for the four N-terminal Ig-like domains of *Drosophila* Dscam (124). Such a large reversal implies considerable flexibility in the segment connecting D2 with D3. In the case of PIB-B, which has two additional membrane-proximal Ig-like domains compared to LILRB, the D4–D5 or D5–D6 connecting segments might provide additional flexibility.

## Ly49 Recognition of a Viral Immunoevasin

Studies of the susceptibility of different mouse strains to infection by MCMV were the first to demonstrate direct recognition of a viral pathogen by NK cells that confers host protection (125). Resistance to infection in C57BL/6 mice is mediated by a single genetic locus in the NKC, which contains both inhibitory and activating Ly49 receptors (126). This locus encodes the activating receptor Ly49H, which impairs MCMV replication (127–130). By contrast, BALB/c mice do not restrict MCMV replication because they lack a gene for Ly49H. Subsequent studies revealed that Ly49H binds directly to a viral glycoprotein, the MHC-I homolog m157, which is expressed on MCMV-infected cells (15, 16). It was also discovered that m157 binds not only to the activating receptor Ly49H in resistant mouse strains, but also to the inhibitory receptor Ly49I in susceptible ones, which explains why MCMV would possess a gene that confers a selective disadvantage to its survival (15, 16, 131).

**Figure 9:**
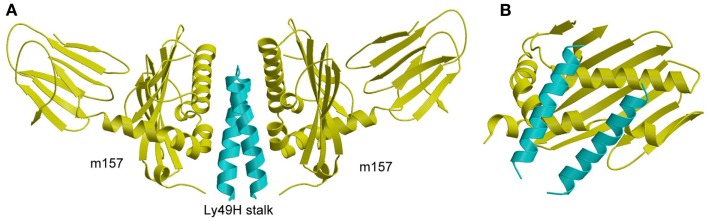
**Structure of m157 bound to the stalk region of Ly49H**. **(A)** Side view of the Ly49H–m157 complex (4JO8), in which two m157 monomers (yellow) engage the Ly49H stalk (cyan). Only part of the helical stalk region of Ly49H (the α3s segment) was visible in electron density. The rest of the Ly49H stalk (segments α1s and α2s) and the NKD were not resolved in the structure. **(B)** Top view of the Ly49H–m157 complex, in which the helical stalks of Ly49H lie across the α1/α2 platform of m157.

The crystal structure of m157 showed that this immunoevasin has an MHC-like fold, although it does not bind peptides or associate with β_2_m (132). Surprisingly, m157 binds to the stalk region of Ly49H, rather than the NKDs, which recognize MHC-I (107). Although m157 was well resolved in the Ly49H–m157 structure, only the α3s segment of Ly49H could be seen in the electron density (Figure 9A). The lack of density for the Ly49H NKD implies considerable flexibility of the NKD in the crystal lattice. In agreement with the structure, solution binding measurements using Ly49H constructs lacking the NKD or stalk region showed that binding to m157 was mediated entirely by the α3s stalk segment, and that the NKD made no appreciable contribution to the interaction (107).

In the complex, two m157 monomers engage the Ly49H dimer, such that the helical stalks lie diagonally across the α1/α2 platform of m157 (Figure 9B). This binding mode is completely distinct from that used by Ly49A and Ly49C to engage MHC-I, whereby the NKDs contact MHC-I at a site beneath the α1/α2 peptide-binding platform (Figures 7A,B). Consequently, m157 does not compete with MHC-I for binding to the NKD. Central to the Ly49H–m157 interaction are two exposed aromatic residues in the Ly49H α3s stalk segment (Tyr115 and Trp123) that make extensive contacts with the immunoevasin. The ability of m157 to target some, but not all, members of the Ly49 receptor family can be correlated with sequence differences in the stalk region (107).

As discussed above, Ly49 receptors can adopt two distinct conformations, backfolded or extended (106). However, the recognition mode observed in the Ly49H–m157 complex only appears possible with Ly49 in the extended state, the conformation that recognizes MHC-I in *cis* (Figure 8B). In the backfolded conformation, by contrast, the Ly49 α3s stalk segment would not be accessible to m157, due to its intimate association with the NKD (Figure 8A). For both the Ly49H–m157 and Ly49I–m157 interactions, kinetic and thermodynamic measurements showed that binding involves a conformational selection mechanism where only the extended conformation of Ly49 is able to bind a first m157 ligand, followed by binding of a second m157 (123). The interaction is characterized by strong positive cooperativity, such that the second m157 binds the Ly49 homodimer 1,000-fold more tightly than the first. The rate-limiting step in the overall mechanism is a conformational transition in Ly49 from its backfolded to extended form.

## Ligand Recognition by NKG2D

NKG2D is a homodimeric C-type lectin-like NK receptor that is expressed on NK cells and cytotoxic T cells. It recognizes multiple structural homologs of MHC-I, including MICA, MICB, ULBP13, and RAE-1β, which all lack a peptide-binding groove and β_2_m (14, 133, 134). ULBP3 and RAE-1β also lack an α3 domain, and are present on the cell surface as glycophosphatidylinositol-linked α1/α2 domains. In humans, expression of MICA and MICB is upregulated in epithelial tumors and stressed cells, compared to little or no expression in normal tissues (135, 136). In rodents, RAE-1, MULT-1, and H-60 are upregulated in tumor cells but not normal cells, similar to MICA and MICB in humans (137, 138). The differential expression pattern of these MHC-related ligands indicates that NKG2D is a key receptor for tumor surveillance by NK cells. In mice, the MCMV-encoded immunoevasins m145, m152, and m155 are involved in downregulating surface expression of the NKG2D ligands MULT-1, RAE-1, and H-60, respectively, thereby thwarting an NKG2D-mediated anti-viral response (63, 139). In humans, the HCMV-encoded immunoevasin UL16 acts as a decoy receptor by binding the NKG2D ligands MICB, ULBP1, and ULBP2 (140). Crystal structures have been reported for human and mouse NKG2D in free form (141, 142), for human NKG2D bound to MICA and ULBP3 (134, 143), and for mouse NKG2D in complex with RAE-1β (144). In addition, structures have been determined for m152 in complex with RAE-1γ (139), and for UL16 bound to MICB (140).

**Figure 10:**
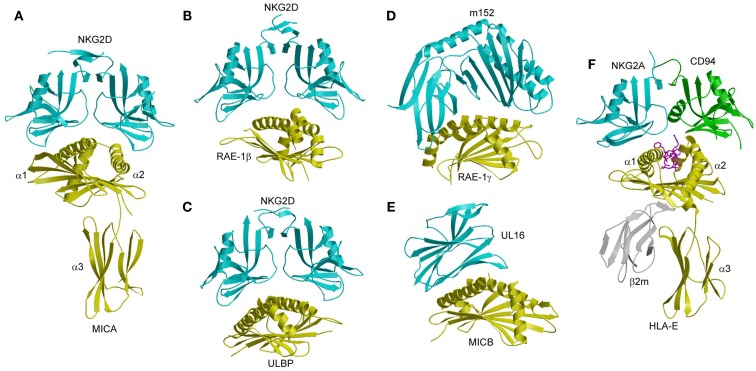
**Structures of NKG2D and NKG2A/CD94 complexes**. **(A)** The human NKG2D–MICA complex (1HYR). The two subunits of the NKG2D homodimer are cyan; MICA is yellow. **(B)** The mouse NKG2D–RAE-1β complex (1JSK). **(C)** The human NKG2D–ULBP3 complex (1KCG). **(D)** Structure of the MCMV immunoevasin m152 bound to the NKG2D ligand RAE-1γ (4G59). **(E)** Structure of the Ig-like HCMV immunoevasin UL16 bound to MICA (2WY3). **(F)** The human NKG2A/CD94–HLA-E complex (3CDG). The NKG2A and CD94 subunits of the NKG2A/CD94 heterodimer are cyan and green, respectively.

MICA consists of an α1/α2 platform domain, which contains the α1 and α2 helices that define the peptide-binding groove in bona fide MHC-I molecules, and a C-type Ig-like α3 domain (Figure 10A) (145). The NKG2D homodimer binds MICA orthogonally to the α1 and α2 helices of the α1/α2 platform (143). This docking mode roughly resembles that of TCR onto MHC-I, but is completely distinct from the way Ly49C binds MHC-I (Figure 7B). Recognition of the asymmetric MICA ligand by the symmetric NKG2D receptor is mediated by similar sites on the NKG2D subunits that contact distinct sites on MICA. In particular, most (7 of 11) contact residues from each NKG2D monomer are shared by both MICA binding sites.

NKG2D binds ULBP3 and RAE-1β orthogonally to the α1/α2 domain of these MHC-like ligands, in a manner resembling the NKG2D–MICA complex (Figures 10B,C) (134, 144). Most notably, a shared subset of NKG2D residues mediates hydrophobic interactions with all three ligands. However, the binding interfaces also display significant differences, such that only one salt bridge and two hydrogen bonds are common to the NKG2D–ULBP3, NKG2D–RAE-1β, and NKG2D–MICA complexes (134, 143, 144).

These structural studies have demonstrated that NKG2D has a remarkable ability to recognize MICA, ULBP3, and RAE-1β using a single binding site, even though these ligands share only ~25% sequence identity. This ability is explained by a rigid adaptation recognition mechanism, rather than induced fit (146). Detailed mutational analysis of NKG2D has shown that the most energetically important residues of the receptor (“hot spots”) interact with relatively conserved residues of MICA, ULBP3, and RAE-1β, without significant conformational changes in NKG2D upon ligand binding (141, 146).

In the MCMV m152–RAE-1γ complex (139), the MHC-I-like immunoevasin binds the α1 and α2 helices of RAE-1γ in a pincer-like manner that resembles the interaction of NKG2D with RAE-1β (Figure 10D). In the HCMV UL16–MICB complex (140), by contrast, the Ig-like UL16 protein uses a three-stranded β-sheet to engage the α1 and α2 helices of MICB, such that residues at the center of the β-sheet mimic a binding motif employed by the structurally unrelated C-type lectin-like NKG2D to bind its diverse ligands (Figure 10E). By competing with NKG2D for ligand binding, m152 and UL16 prevent NKG2D-mediated NK cell activation and thus promote viral survival (147–149).

## Recognition of HLA-E by NKG2/CD94 Receptors

In addition to NKG2D, which exists as a homodimer on the NK cell surface, the NKG2D family includes NKG2A, NKG2B, NKG2C, and NKG2E, all of which form obligate heterodimers with CD94 (150–152). NKG2A and NKG2B contain ITIM motifs in their cytoplasmic tails and function as inhibitory receptors; NKG2C and NKG2E associate with the ITAM-containing DAP12 molecule and are activating receptors. The ligand for NKG2/CD94 receptors is the non-classical MHC-I molecule HLA-E, which binds a restricted set of peptides derived from the leader peptides of classical and non-classical MHC-I proteins (150–152). Because HLA-E does not express on the cell surface without a bound peptide, HLA-E expression depends on the production of other MHC-I molecules. Therefore, recognition of HLA-E by NKG2/CD94 receptors enables NK cells to monitor the expression of other HLA class I proteins on cells. This double-check mechanism ensures that cells are producing MHC-I molecules in a normal manner.

The crystal structure of NKG2A/CD94 has been determined in unbound form (153), and in complex with HLA-E bound to a peptide derived from the leader sequence of HLA-G (154, 155). In the complex, NKG2A/CD94 straddles the peptide-binding cleft of HLA-E, with the NKG2A and CD94 subunits mainly interacting with the α2 and α1 helices of HLA-E, respectively (Figure 10F). No significant conformational changes in NKG2A/CD94 or HLA-E occur upon complex formation, indicating a lock-and-key binding mechanism, as in the case of NKG2D (141, 146).

Most (~70%) of the buried surface area in the NKG2A/CD94–HLA-E complex is contributed by the invariant CD94 subunit (154, 155). Thus, CD94 dominates the interaction with HLA-E, whereas NKG2A is more peripheral to the interface. The peptide accounts for ~20% of the buried surface area on the HLA-E side of interface, in which CD94 again dominates the interactions with peptide, albeit with poor shape and chemical complementarity (154). CD94 is positioned over the P8 residue of the peptide, with additional contacts to residue P5. The focus of NKG2A/CD94 on the C-terminal half of the peptide is notable, since nearly all of the limited sequence variation among HLA-E-restricted peptides is concentrated in the C-terminal residues, which are read out primarily by the invariant CD94 subunit.

In sharp contrast to the dominant role of hydrophobic interactions in ligand recognition by NKG2D (134, 144), the NKG2A/CD94–HLA-E interface is mostly electrostatic in nature (154). The interface is characterized by a large number of polar interactions, including 19 hydrogen bonds and 8 salt bridges. This helps explain the fidelity of NKG2A/CD94 for HLA-E compared to the promiscuity of NKG2D for multiple ligands, as discussed above.

## Cadherin Recognition by KLRG1

Killer cell lectin-like receptor G1 is a C-type lectin-like inhibitory receptor that contains an ITIM motif in its cytoplasmic region (156, 157). KLRG1 is found on 50–80% of human NK cells, and its expression is highly upregulated following infection with viruses or parasites (158–161). The biological ligand for KLRG1 is E-cadherin (32, 33, 162). E-cadherin, whose extracellular region comprises five Ig-like domains (EC1–EC5), is localized at the basolateral membrane of epithelial cells where it establishes tight binding between neighboring cells in adherens junctions (163). Besides E-cadherin, KLRG1 recognizes N- and R-cadherins (32), which are present in analogous structures in other cell types. The binding of E-cadherin to KLRG1 prevents lysis of E-cadherin-expressing epithelial cells by KLRG1^+^ NK cells, thereby preventing tissue damage (32, 71, 164). In addition, KLRG1 may play a role in tumor immunosurveillance analogous to missing self-recognition by inhibitory NK receptors that bind MHC-I (Ly49s and KIRs) (164, 165). Because the malignancy of epithelial tumors is frequently associated with down-regulation of E-cadherin, the KLRG1–E-cadherin system may serve to detect potentially metastatic tumors with abnormal cadherin expression (71, 164, 166).

**Figure 11:**
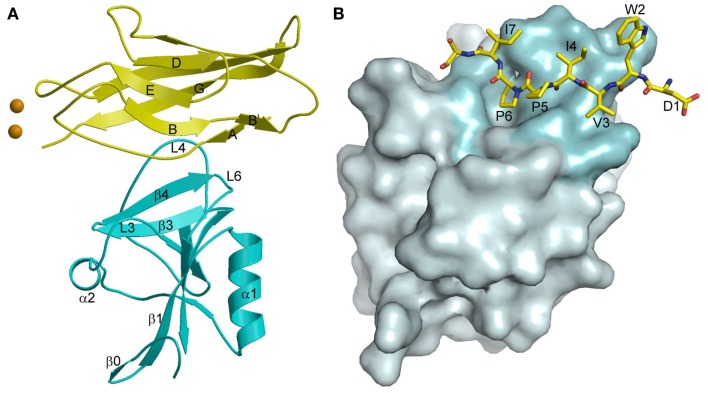
**Cadherin recognition by KLRG1**. **(A)** Structure of KLRG1 in complex with the membrane-distal D1 domain of E-cadherin (3FF8). KLRG1 is cyan and E-cadherin is yellow. Secondary structure elements are labeled. Bound Ca^2+^ ions are drawn as brown spheres. **(B)** The KLRG1–E-cadherin binding interface. The KLRG1 molecular surface is shown in gray with the region contacting E-cadherin colored cyan. Residues 1–8 of E-cadherin are drawn in stick format and labeled.

In the crystal structure of the complex between KLRG1 and the EC1 domain of E-cadherin, one KLRG1 CTLD binds one EC1 molecule (Figure 11A) (167). In this respect, KLRG1 recognition of its non-MHC ligand is reminiscent of Ly49 recognition of MHC-I, in which each CTLD monomer contains an entire ligand-binding site (Figure 7B). By contrast, the binding site of NKG2D for the MHC-related ligand MICA (143) (Figure 10A), as well as the binding site of NKG2A/CD94 for HLA-E (154, 155) (Figure 10F), is formed by two CTLD subunits. E-cadherin docks onto a surface of KLRG1 that roughly corresponds to the ligand-binding site of Ly49s and other C-type lectin-like NK receptors (167). This site is formed by three loops (L3, L4, and L6) and β-strand 4 (Figure 11A), which interact primarily with residues Val3–Ile7 of E-cadherin (Figure 11B). These five residues are absolutely conserved in E-, N-, and R-cadherins, which enables NK cells bearing a single KLRG1 receptor to monitor expression of multiple cadherins on target cells, resulting in MHC-independent missing self-recognition.

The KLRG1–E-cadherin complex buries a total solvent-accessible surface of only 1140 ^2^. This exceptionally small interface is at the lower limit of the average value of 1600 (±400) Å^2^ for stable protein–protein complexes (168), and likely explains the relatively low affinity of the KLRG1–E-cadherin interaction (*K*_D_ = 150 μM), which it is considerably weaker than for any other NK receptor–ligand pair characterized to date (167). KLRG1 may compensate for its exceptionally low monomeric affinity for cadherins through multipoint attachment to cadherin molecules on the target cell surface. Additionally, the ability of KLGR1 to form disulfide-linked dimers (169), or even multimers (170), may further increase the avidity of KLRG1–cadherin interactions. In this way, KLRG1–cadherin recognition could be achieved through the cooperativity of multiple associations, rather than by relying on the stability of individual complexes, while still allowing for dissociation of the complexes during transient NK cell–target cell encounters.

## Genetically Linked C-Type Lectin-Like Receptor–Ligand Pairs

The NKC encodes approximately 30 type II transmembrane glycoproteins that are members of the C-type lectin-like superfamily (171). NKC genes are divided into killer cell lectin-like receptor (KLR) genes and C-type lectin receptor (CLEC) genes. KLR genes code for molecules expressed on NK cells. CLEC genes code for molecules expressed on other cell types, such as dendritic (CLEC9A) and myeloid (CLEC2B) cells.

The KLR family includes Ly49, NKG2D, and CD94/NKG2A receptors that bind MHC-I or MHC-I-like molecules, as discussed above. The KLR family also includes receptors that recognize non-MHC ligands. This category includes KLRG1, which binds E-cadherin (167) (Figure 11A), in addition to receptors that bind CLEC2 proteins which themselves belong to the C-type lectin-like superfamily (21). The genes encoding these KLR–CLEC2 receptor–ligand pairs are genetically linked in the NKC. For example, in mice, the inhibitory KLR family member receptor Nkrp1d binds Clrb (172, 173). Down-regulation of Clrb expression by genotoxic stress or tumorigenesis triggers NK-cell-mediated lysis, supporting the concept of MHC-independent control of NK cell function by Nkrp1 receptors (173, 174). In humans, the inhibitory NK receptor NKR-P1A binds the CLEC2 family member LLT1, reducing NK-cell-mediated cytotoxicity and interferon-γ secretion (175–177). Viral induction of LLT1 expression in B cells points to a role for the NKR-P1A–LLT1 interaction in modulating immune responses to pathogens (178). The human activating NK receptor NKp80 recognizes the CLEC2 family member AICL, which is genetically coupled to NKp80 in the NKC (21, 31). The NKp80–AICL pair promotes cross-talk between NK cells and monocytes (31). In addition to monocytes, AICL is expressed on monokine-activated human NK cells that also express NKp80, which may enable autonomous control of NK cell responses (179).

Keratinocyte-associated C-type lectin (KACL) is a newly identified member of the human CLEC2 family (180). Notably, KACL is expressed almost exclusively in the skin. KACL is a ligand for the activating receptor NKp65, which is genetically linked to KACL in the NKC (29). Upon binding KACL on keratinocytes, NKp65 triggers NK-mediated cytotoxicity and proinflammatory cytokine release. Thus, the NKp65–KACL interaction may contribute to the immunosurveillance of human skin (21, 29, 181).

**Figure 12:**
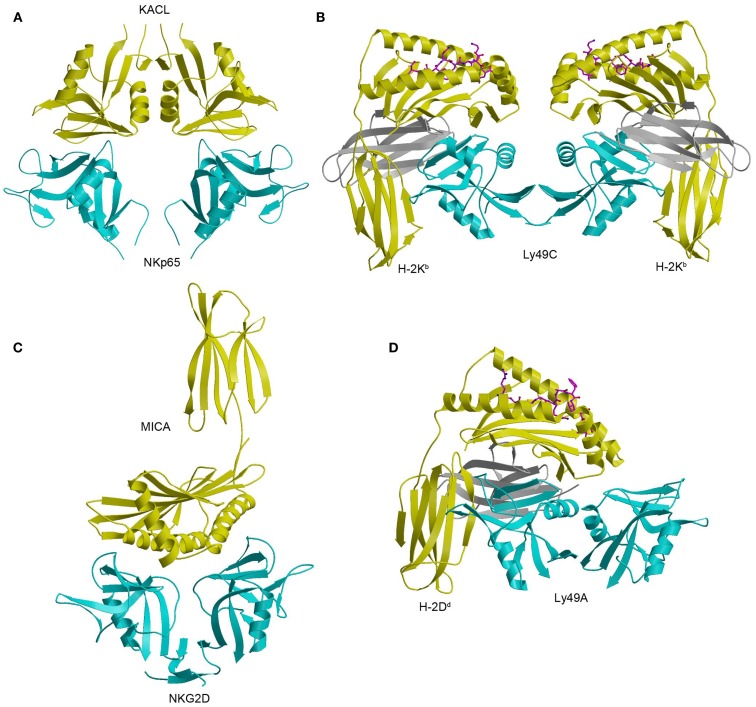
**Structure of the NKp65–KACL complex and comparison with other NKC-encoded receptor complexes**. **(A)** Structure of the human NKp65–KACL complex (4IOP). NKp65 is cyan and the KACL dimer is yellow. **(B)** Structure of the Ly49C–H-2K^b^ complex (3C8K). The Ly49C dimer is cyan, the H-2K^b^ heavy chain is yellow, and β_2_m is gray. **(C)** Structure of the NKG2D–MICA complex (1HYR). The NKG2D dimer is cyan and MICA is yellow. **(D)** Structure of the Ly49A–H-2D^d^ complex (1QO3). The Ly49A dimer is cyan, the H-2D^d^ is heavy chain is yellow, and β_2_m is gray.

The structure of NKp65 bound to KACL has revealed the basis for genetically coupled recognition in the NKC (182). KACL forms a homodimer similar to the NKG2D and Ly49 homodimers; NKp65, contrast, is monomeric (Figure 12A). KACL binds NKp65 bivalently, in a manner resembling the Ly49C–H-2K^b^ complex (Figure 12B) (103, 104), except that, in the NKp65–KACL complex, it is the ligand (KACL), instead of the receptor (NKp65) that is dimeric. This bivalent binding mode is completely different from those employed by the dimeric NKG2D and Ly49A receptors. Thus, the NKG2D dimer engages one MICA molecule using a single binding site formed by the association of its two subunits (Figure 12C), whereas the Ly49A dimer binds a single H-2D^d^ ligand using only one subunit (Figure 12D).

In the NKp65–KACL complex (182), two C-type lectin-like proteins engage each other in a head-to-head orientation utilizing similar structural elements: NKp65 uses loops L0, L3, L5, and L6 and strands β3 and β4 to contact the analogous loops and strands of KACL (Figure 12A). A mutational analysis of KACL residues in contact with NKp65 showed that all hotspot residues of KACL are conserved or conservatively substituted in AICL and LLT1, and that these hotspot residues contact residues on NKp65, NKp80, and NKR-P1A that are themselves conserved (182). Therefore, the docking mode observed in the NKp65–KACL complex also applies to other NKC-encoded receptor–ligand pairs, including NKp80–AICL, NKR-P1A–LLT1, and Nkrp1–Clr.

NKp65 binds KACL with exceptionally high affinity (*K*_D_ = 6.7 × 10^−10^ M), compared to other cell–cell recognition molecules, whose *K*_D_s are generally micromolar (183). Indeed, the affinity of NKp65 for KACL is 70,000-fold higher than that of NKR-P1A for LLT1 (184) and 3,000-fold higher than that of NKp80 for AICL (31). In contrast to NKR-P1A and NKp80, which exist as disulfide-linked dimers, NKp65 is not disulfide-linked on the NK cell surface. Likewise, AICL and LLT1 (21, 176, 177, 181), but not KACL (29), form disulfide-linked dimers on cells. Dimerization of NKp80 and NKR-P1A may compensate for the low (micromolar) affinities of these receptors, relative to NKp65, by increasing avidity via bivalent binding of their AICL and LLT1 ligands, which are themselves dimeric. By contrast, the high (nanomolar) affinity of the NKp65–KACL interaction may overcome the need for receptor dimerization by generating complexes of half-life comparable to those of the NKp80–AICL and NKR-P1A–LLT1 complexes, resulting in efficient signaling.

## Future Directions

The structural studies described in this review have enabled us to understand how representative NK receptors recognize cellular and viral ligands at the atomic level. However, the biophysical mechanisms by which inhibitory or activating signals are transmitted to the NK cell following ligand engagement remain largely a mystery. It is also unknown how inhibitory and activating signals are integrated within the NK cell to ultimately determine the outcome of NK cell–target cell encounters.

Only recently have structural studies begun to elucidate the molecular details of the signal transduction process. Crucial for NK cell triggering is the association of the transmembrane region of activating NK receptors, such as NKG2D and NKp44, with ITAM-bearing signaling molecules, such as DAP10 and DAP12. NMR has been used to determine the structure of the heterotrimeric assembly formed by the transmembrane regions of NKG2C and DAP12 (185). The main contact site comprises an intricate electrostatic network involving five hydrophilic transmembrane residues: two aspartates and two threonines from the DAP12 dimer that together interact with a lysine from NKG2C. Such studies of membrane-embedded NK receptors, and their association with signaling proteins, promise to provide critical information for linking ligand recognition to NK cell activation or inhibition.

## Conflict of Interest Statement

The authors declare that the research was conducted in the absence of any commercial or financial relationships that could be construed as a potential conflict of interest.
